# Physiologically Based Pharmacokinetic Modelling to Predict Imatinib Exposures in Cancer Patients with Renal Dysfunction: A Case Study

**DOI:** 10.3390/pharmaceutics15071922

**Published:** 2023-07-11

**Authors:** Karen Rowland Yeo, Oliver Hatley, Ben G. Small, Trevor N. Johnson

**Affiliations:** Certara UK Limited, Simcyp Division, Level 2-Acero, 1 Concourse Way, Sheffield S1 2BJ, UK; oliver.hatley@certara.com (O.H.); ben.small@certara.com (B.G.S.); trevor.johnson@certara.com (T.N.J.)

**Keywords:** renal impairment, cancer, PBPK modelling, inclusivity

## Abstract

Imatinib is mainly metabolised by CYP3A4 and CYP2C8 and is extensively bound to α-acid glycoprotein (AAG). A physiologically based pharmacokinetic (PBPK) model for imatinib describing the CYP3A4-mediated autoinhibition during multiple dosing in gastrointestinal stromal tumor patients with normal renal function was previously reported. After performing additional verification, the PBPK model was applied to predict the exposure of imatinib after multiple dosing in cancer patients with varying degrees of renal impairment. In agreement with the clinical data, there was a positive correlation between AAG levels and imatinib exposure. A notable finding was that for recovery of the observed data in cancer patients with moderate RI (CrCL 20 to 39 mL/min), reductions of hepatic CYP3A4 and CYP2C8 abundances, which reflect the effects of RI, had to be included in the simulations. This was not the case for mild RI (CrCL 40 to 50 mL/min). The results support the finding of the clinical study, which demonstrated that both AAG levels and the degree of renal impairment are key components that contribute to the interpatient variability associated with imatinib exposure. As indicated in the 2020 FDA draft RI guidance, PBPK modelling could be used to support an expanded inclusion of patients with RI in clinical studies.

## 1. Introduction

The US Food and Drug Administration (FDA) 2010 draft guidance document [1] indicated that the assessment of the pharmacokinetics (PKs) of drugs in patients with impaired renal function (RF) should be conducted for most small molecule drugs intended for chronic use, irrespective of their elimination pathways. On September 4, 2020, the FDA published a revised draft guidance which was made available for public comments prior to its finalization [2]. Along with the current European Medicines Agency (EMA) 2015 guideline [3], this revised draft of the FDA Guidance [3] describes the approaches that pharmaceutical companies should consider when developing strategies for assessing the impact of varying degrees of RI (mild, moderate and severe) on the PK of drugs in development. With respect to other global regulatory authorities, it appears that the Pharmaceutical and Medicines Devices Agency from Japan and the National Medical Products Administration from China support the FDA Guidance while the Therapeutic Goods Administration from Australia has formally adopted the EMA guidelines [4].

The main objective of conducting an RI study is to inform a dose recommendation for the specific population on the drug label. If RI is expected to significantly alter the PK of the drug or metabolite, i.e., renal excretion is the main route of elimination for the drug or metabolite, a clinical PK study in patients with RI is recommended. A reduced clinical study design can be applied initially in non-dialyzed end-stage renal disease (ESRD) or patients with severe RI for drugs mainly cleared by nonrenal routes. Depending on the results, further investigation may be required in other RI classes. The absorption, distribution, metabolism and excretion (ADME) of drugs can change because of accumulated uremic toxins which affect specific processes such as hepatic metabolism, transporter-mediated uptake and efflux, and protein binding [5,6]. The proposed mechanisms have clinical relevance in patients with RI and have been discussed in detail by Nolin et al. [7].

Typically, a single-dose RI study is conducted if the drug in development displays linear kinetics, and the same dose is given in both the RI and control groups. Although a multiple-dose study is more appropriate for drugs with non-linear or time-dependent PK, it is recognised that careful consideration must be given to patient groups with a higher prevalence of RI such as those with cancer. Despite this, neither the current FDA guidance (2010) nor the updated guidance (2020) provide clear strategic or decision pathways for RI studies in oncology patients [1,3]. According to a recent review of new drug approvals (NDA) for oncology drugs [8], it was concluded that the most appropriate strategy for carrying out a RI study during clinical development or as a post-marketing study requires consideration of the totality of evidence with reference to the estimated therapeutic window. In each of the NDA reviews, population PK (PopPK) analysis was used to assess the impact of RI on PK and was typically carried out when limited, or no, data were available from a dedicated RI study to complement the findings from the mass balance studies. This approach is recommended in the revised 2020 FDA guidance document [3].

High-fidelity PBPK models with a sufficient mechanistic resolution of in vivo human clearance routes (e.g., biliary, renal and metabolic), are primed for assessing the impact of RI on drug PK in development [9]. Indeed, the International Consortium for Innovation and Quality in Pharmaceutical Development predicted the effects of RI on drug exposure across 50 study arms [10]; verified PBPK models were available for 25 compounds mainly eliminated by cytochrome P450 (CYP) enzymes with varying contributions of biliary excretion and renal clearance (20% ≥ 25% renally excreted). The finding that 64% of predictions were within 1.25 fold of observed data and 84% within 1.5 fold supports the application of PBPK models for assessment of RI. Recognising that this mechanistic approach is entirely suited to this purpose, early use of PBPK models to support an expanded inclusion of patients with RI in clinical studies has been highlighted by the FDA in the 2020 FDA draft RI guidance [3].

Key changes in physiological parameters reflecting the effects of RI include reductions of GFR (elevated serum creatinine), inhibition of CYP enzyme or transporter activities (circulating uremic toxins) and a decrease in albumin and an increase in α-acid-glycoprotein (AAG) levels. In cases where a drug binds mainly to albumin in plasma, the increased unbound fraction often reported for patients with renal dysfunction can attenuate the effects of the reduction of CYP activity on total drug exposures; although higher concentrations of the drug are available for uptake into the liver, less metabolism may occur due to the inhibited CYP activity. While AAG represents a relatively small portion (~1–3%) of the total plasma proteins, compared to ~60% composition of albumin, it can still play a significant role in drug binding and PK [11]. Since AAG levels increase in most disease states including RI and cancer, drugs with a high affinity may demonstrate significantly higher binding (lower unbound fraction) which can augment the effects of the reduced CYP activity leading to larger than expected increases in total drug exposure. 

Imatinib, an anticancer drug and more specifically a tyrosine kinase inhibitor, was approved by the FDA for the treatment of chronic myelogenous leukemia (CML) and gastrointestinal stromal tumors (GIST). As a consequence of its high binding affinity to AAG and its ~55 fold weaker binding affinity to albumin, a non-linear relationship exists between the free fraction of imatinib and total plasma concentrations [12]. Elevated levels of AAG in patients have been associated with a delayed or lack of response to imatinib treatment; accounting for differences in plasma protein binding can lead to the selection of a dose with improved efficacy in patients [13,14]. A PBPK model for imatinib was reported previously [15] and was verified using clinical PK data from healthy and GIST and CML populations with normal renal function and in paediatrics. In this study, we have extended the application of a PBPK modelling approach to predict drug PK in patients with advanced malignancies and varying degrees of renal dysfunction and to elucidate the impact of comorbidities in a cancer population, using imatinib as an illustrative example. 

## 2. Methods

### 2.1. Software

The Simcyp population-based PBPK Simulator (Version 21 Release 1; Certara UK Limited, Sheffield, UK) was used to generate plasma concentration-time profiles of imatinib in healthy subjects and cancer patients with varying degrees of renal impairment. The default SV-ketoconazole file was used in simulations of the drug interaction with imatinib. Clinical study data from the literature were digitized with GetData Graph Digitizer (version 2.22, http://getdata-graph-digitizer.com, accessed on 24 April 2023). Unless specifically stated, default population files were used in simulations.

### 2.2. Reported Clinical Data

Fourteen healthy subjects (13 male, 1 female) aged 35–59 years and weighing 64–103 kg were recruited into a drug interaction study between imatinib and ketoconazole [16]. Subjects received a single oral dose of imatinib 200 mg alone and a single oral dose of imatinib 200 mg co-administered with a single oral dose of ketoconazole 400 mg according to a two-period crossover design. Blood samples for determination of plasma imatinib concentrations were obtained up to 96 h after dosing.

Data from 49 GIST patients (51% female) aged 25 to 88 years with acceptable renal function (CrCL ≥ 60 mL/min), receiving imatinib at daily oral doses ranging from 200 to 800 mg were collected over a period of 2 years [17]. Most blood samples were drawn at 1–6-month intervals on follow-up visits as part of an observational clinical PK study. The median number of measurements for each patient was 3 (range 1–11) and was obtained under steady-state conditions. Observed concentrations in patients at steady state were scaled for 400 mg once daily dosing. 

Sixty cancer patients (52% female) aged 16 to 84 years with advanced malignancies and varying degrees of renal dysfunction were recruited into a clinical study to determine the safety, dose-limiting toxicities, MTD, and pharmacokinetics of imatinib and to develop dosing guidelines for imatinib in such patients [18]. Fourteen patients were entered into group A (normal renal function, creatinine clearance (CrCL) ≥ 60 mL/min), 22 patients were entered into group B (mild dysfunction, CrCL 40 to 59 mL/min), 22 patients were entered into group C (moderate, CrCL 20 to 39 mL/min) and two patients were entered in group D (severe, CrCL < 20 mL/min). The patients received daily imatinib doses of 100 to 800 mg (Table 1). An average daily dose was estimated for each group weighted using the number of patients on each dose (Table 1). Pharmacokinetic sampling was performed on the first day and day 15 after initiation of treatment. Although no concentration-time profiles were available, dose normalised PK parameters were.

### 2.3. Virtual Populations

Derivation of a virtual North European Caucasian population (default values for physiological parameters including liver volume, blood flows, and enzyme abundances) has been described in detail previously [19]. The Sim-Cancer population was developed previously using this population as the baseline, and data from solid tumour patients were included wherever possible; parameters relating to this population have been described in detail [20]. In summary, key changes in physiological parameters reflecting the effects of cancer include reductions of GFR (elevated serum creatinine), a decrease in albumin, and an increase in AAG levels. Following analysis of relevant data in the literature, no changes in CYP enzyme expression were included relative to the baseline population. For simulations in cancer patients, the mean plasma concentrations of AAG were modified from the default values of 1.48 g/L (coefficient of variation [CV] 34.3%) to AAG values recorded in the actual clinical studies [17,18]. The default female-to-male ratio in AAG concentrations of 0.9 was maintained throughout the simulations. For cancer patients with normal renal function, a value of 1.06 (CV-50%) was used for males. For cancer patients with mild and moderate renal function, values of 1.77 (CV-50%) and 1.64 g/L (CV-39%), respectively, were used.

In the cancer study [18], patients were assigned to normal renal function (CrCL ≥ 60 mL/min), mild dysfunction (CrCL 40 to 59 mL/min), moderate dysfunction (CrCL 20 to 39 mL/min) and severe dysfunction (CrCL < 20 mL/min) categories. Three RI populations are available within the Simcyp Simulator; mild (CrCL 60 to 89 mL/min), moderate (CrCL 30 to 59 mL/min) and severe (CrCL 15 to 29 mL/min). Key changes in physiological parameters reflecting the effects of RI include reductions of GFR (elevated serum creatinine), reductions of CYP enzyme abundances, a decrease in albumin and an increase in AAG. Although the populations were not used directly in simulations, changes in CYP3A4 enzyme expression because of RI (values of 118, 95.2 and 87.3 pmol/mg microsomal protein for mild, moderate and severe RI versus 137 in healthy adults with normal renal function) [21,22] were evaluated in simulations of cancer patients with varying degrees of renal dysfunction. Although changes in CYP2C8 are not currently accounted for in the RI populations within the Simcyp Simulator, published data indicate that CYP2C8 model drugs showed a consistent decrease in unbound CYP2C8-mediated clearance with increasing severity, with an average of 39% reduction within the severe RI group [6]. Given that the moderate RI category within the cancer study (CrCL 20 to 39 mL/min) appears to be a combination of the moderate (CrCL 30 to 59 mL/min) and severe RI (CrCL 15 to 29 mL/min) categories implemented within the Simcyp Simulator, a CYP2C8 abundance of 14.6 pmol/mg protein (39% reduction from the baseline of 24) was applied in simulations.

### 2.4. Imatinib PBPK Model

A PBPK model for imatinib published previously was used for the simulations [15]. Drug-related input parameters and key assumptions relating to the development of the model were described in detail; a summary is provided here. As a basic compound, imatinib binds extensively to AAG [12]. The unbound fraction in plasma (fup) of 0.05 used for imatinib was based on the reported value in healthy European populations. Within the Simcyp Simulator, generated AAG levels ([P]) for a cancer population are used against a reference value assigned for healthy subjects ([P]_pop_) to calibrate the fu (fu_pop_) from a healthy population value (fu) to that of a disease population, according to Equation (1) [23].

(1) $$fu=\frac{1}{1+\left[\frac{\left[P\right]}{{\left[P\right]}_{pop}}\times \frac{\left(1-fu_{pop}\right)}{fu_{pop}}\right]\;}$$

It should be noted that this equation only accounts for changes in fu due to changes in AAG levels and does not consider changes in the binding affinity of a drug that may occur due to accumulated uremic toxins [17]. Furthermore, a value is calculated for each virtual individual based on their assigned AAG levels which are generated from the mean and CV that are used as inputs for each of the populations. Only unbound drug is available for uptake into the liver to undergo transformation. The conversion of imatinib to N-desmethyl imatinib and other metabolites via CYP3A4 and CYP2C8 based on in vitro data is also considered within the model along with an undefined metabolic component that is required to scale the in vitro data to a clearance that is consistent with observed data. Mechanism-based inhibition (MBI) of CYP3A4 is also integrated within the model and auto-inhibition occurs during multiple dosing. 

### 2.5. Simulations

The demographic (including age and gender) characteristics and dose regimen used in the simulations were matched to the clinical studies and the number of virtual subjects was based on 10 trials of the number of subjects used in the corresponding clinical study. 

In order to verify the contribution of CYP3A4 to the base model (prior to auto-inhibition), we assessed the DDI with ketoconazole in healthy subjects. It should be noted that this had not been done or reported previously for the initial model development [15]. Ten virtual trials of 14 healthy subjects (13 male, 1 female) aged 35–59 years and aged 18 to 44 years receiving a single oral dose of imatinib 200 mg co-administered with a single oral dose of ketoconazole 400 mg were generated, and the simulated and observed plasma concentrations [16] and pharmacokinetics of imatinib were compared. 

Thereafter, we ran simulations of GIST patients with normal renal function where observed plasma concentration data were available for verification [17]. The model developed previously [15] was verified using single-dose PK data in healthy subjects and multiple-dose PK data in GIST and CML patients with normal renal function. Given that the simulations were conducted using the North European Caucasian population and that only the AAG levels were changed to represent GIST and CML populations (despite the different age–sex distribution versus healthy), we performed some additional verification of the imatinib PBPK model using the Sim-Cancer population. Ten virtual trials of 14 cancer patients (51% female) aged 25 to 88 years receiving daily oral doses of 400 mg were generated and the simulated and observed plasma concentrations of imatinib were compared. 

It should be noted that the combination of the elevated AAG levels with the baseline value fu of 0.05 (in healthy adults) led to a fu that was significantly lower than the measured value in GIST patients (0.062). Significant interlaboratory variability has been reported previously for measurements of the unbound fraction of imatinib [12]. For calibration purposes, the baseline value was increased to 0.07 in patients with normal renal function; this allowed recovery of the observed fu (0.062) using the relevant AAG level in GIST patients. Going forward, this value (0.07) was applied in all simulations involving cancer patients and their respective AAG levels to allow recovery of the observed fu values in the clinical study.

Finally, simulations were run in cancer patients (52% female) aged 16 to 84 years with varying degrees of renal dysfunction. Simulations were not performed for severe RI due to the small number of patients recruited into the clinical study (n = 2). Ten virtual trials of 14 cancer patients (normal renal function) receiving daily oral doses of 629 mg for 15 days, 22 cancer patients (mild renal dysfunction) receiving daily oral doses of 645 mg for 15 days and 22 cancer patients (moderate renal dysfunction) receiving daily oral doses of 418 mg were generated and the simulated and observed PK of imatinib [18] were compared. In each of these three sets of simulations, apart from the AAG levels, the Sim-Cancer population was applied initially without any modification. Thereafter, changes in CYP enzymes to reflect the impact of RI were applied for patients with moderate renal dysfunction. Firstly, simulations were run using reduced levels of CYP3A4 only and were then repeated using reduced levels of both CYP3A4 and CYP2C8 to determine whether there was improved recovery of the observed data.

## 3. Results

### 3.1. Verification of Imatinib PBPK Model

**Figure 1:**
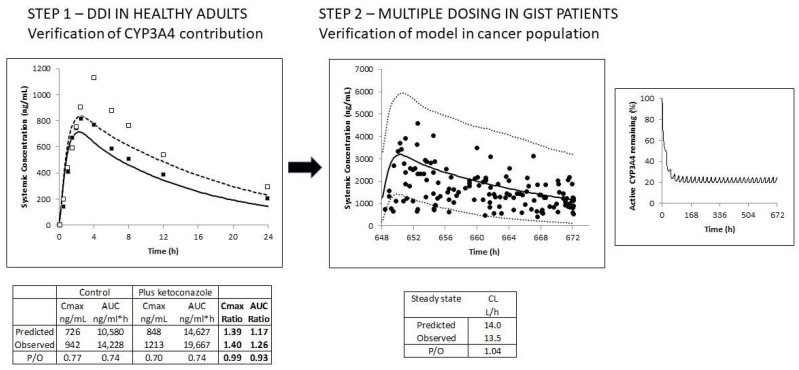
The schematic illustrates the verification of the imatinib PBPK model. Step 1: The contribution of CYP3A4 to the clearance of imatinib was verified using the clinical DDI study with ketoconazole in healthy volunteers. Simulated (lines) and observed (data points) total plasma concentration-time profiles of imatinib (200 mg) in the absence (solid line; black squares) and presence of ketoconazole (400 mg) (dashed line; open squares) are shown. Step 2: Then, the model was verified in GIST patients following multiple oral daily doses of 400 mg. Simulated (black line) and observed (data points) total plasma concentration-time profiles of imatinib are shown, along with the 5^th^ and 95^th^ percentiles of the total virtual population (dashed lines). Active CYP3A4 activity is significantly reduced during multiple dosing of imatinib due to autoinhibition of CYP3A4-mediated metabolism (right-hand figure).

Mean simulated and observed plasma concentrations of imatinib following a single oral dose of 200 mg in the absence of and co-administered with a single dose of ketoconazole (400 mg) are shown in Figure 1. The predicted change in the exposure of imatinib was consistent with observed data (within-1.25-fold) as indicated by the C_max_ and AUC ratios (Figure 1), thus confirming that assignment of approximately 40% of the metabolism to CYP3A4 is appropriate at baseline. 

After multiple oral doses of 400 mg imatinib in GIST patients, accumulation of the drug occurred; the simulations indicated that only 20% of the assigned CYP3A4-mediated metabolism (40%) remained at a steady state due to autoinhibition of this pathway (Figure 1). Despite the significant variability, the observed data were captured reasonably well using the virtual cancer population (visual inspection). Indeed, more than 90% of the total drug concentrations fell within the prediction interval (5^th^ to 95^th^ percentile of the PBPK model predictions), as shown in Figure 1. Furthermore, the predicted mean CL/F at steady state was consistent with the reported value; values were 14.0 versus 13.5 L/h, respectively.

### 3.2. Prediction of Imatinib Exposures in Cancer Patients with Varying Renal Impairment

**Figure 2:**
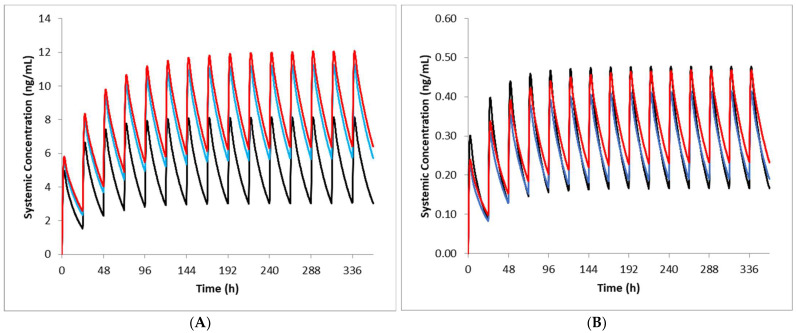
Simulated imatinib total (**A**) and unbound (**B**) plasma concentration-time profiles in cancer patients with varying renal function. Dose normalised simulated concentration-time profiles of imatinib following oral daily dosing of 629 mg, 645 mg 418 mg in patients with normal (black line), mild RI (blue line) and moderate RI (red line) are shown for 14 days (**A**) and on the last day of dosing (**B**). In the moderate RI patients, reduced hepatic CYP3A4 and CYP2C8 abundances were applied.

Using the AAG levels measured clinically for each group, predicted fu values were 0.062, 0.042 and 0.042 for cancer patients with normal renal function, mild renal dysfunction and moderate renal dysfunction, respectively. Corresponding observed values were 0.062, 0.061 and 0.047. Predicted plasma total exposures of imatinib were within 1.25 fold of observed data in cancer patients with normal renal function and mild RI after multiple oral doses of imatinib (Table 2). Although the predicted exposures also appeared to be reasonably well predicted for patients with moderate RI (Table 2), this was not the case for the relative change in exposure when compared to patients with normal renal function (Table 3). Thus, the simulations were repeated initially using a lower hepatic CYP3A4 abundance and then lower CYP3A4 and CYP2C8 abundance values simultaneously. CYP reductions to reflect changes in expression for patients with moderate RI led to improved predictions (Table 2). Although the CYP changes did not appear to have a significant impact on the predicted imatinib exposures at steady state (Figure 2), there was an improvement in the relative change in total exposure when compared against patients with normal renal function (Table 2); predicted versus observed AUC ratios were 1.43 versus 2.01 (no CYP changes) and 1.71 versus 2.01 (CYP changes). Not surprisingly, for the cancer patients with mild renal dysfunction, where the fu was underpredicted compared with the observed value, the unbound exposures of imatinib were less well predicted than in the other categories.

**Figure 3:**
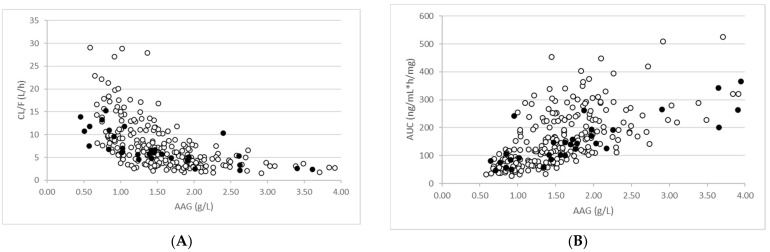
Relationship between AAG levels and total imatinib exposure (**A**) and clearance (**B**). Simulated (open circles) and observed (closed circles) are shown for patients with mild and moderate RI.

Consistent with the observed data, there was a positive correlation between AAG concentrations and total dose-normalised imatinib AUC on day 15 and an inverse correlation between AAG concentrations and total imatinib CL/F (Figure 3). The simulations support the significant impact that AAG has on the PK of imatinib in cancer patients with elevated AAG levels. It should be noted that when unbound concentrations of imatinib were applied, there was no correlation between AAG levels and AUC or CL/F.

## 4. Discussion

A PBPK model for imatinib describing the CYP3A4-mediated autoinhibition during multiple dosing in GIST patients with normal renal function was reported previously [15]. After additional verification was conducted in this study, the model was applied to predict the exposure of imatinib after multiple dosing in cancer patients with varying degrees of renal impairment. In agreement with the clinical data [18], there was a positive correlation between AAG levels and imatinib exposure (Figure 3A). The increased AAG levels lead to lower unbound fractions in the plasma which then reflects in a lower clearance (Figure 3B). Of particular note was the finding that to recover the observed data in cancer patients with moderate RI (CrCL 20 to 39 mL/min), reductions of hepatic CYP3A4 and CYP2C8 expression, which reflect the effects of RI, had to be included in the simulations. This did not appear to be the case for mild RI (CrCL 40 to 50 mL/min). It is important to note that in addition to being altered by the increased AAG levels in cancer patients with renal dysfunction, the fu of imatinib could also be affected by a change in the AAG binding affinity of the drug [17] due to competition from circulating uremic toxins which accumulate in patients with renal dysfunction. One of the key limitations of our study is that this was not considered in the simulations. Despite this, fu values for the cancer patients with normal renal function and moderate renal dysfunction were accurately predicted when based on AAG levels in these patients alone.

For drugs of intermediate to low hepatic extraction with a high degree of protein binding such as imatinib, a change in plasma AAG levels or AAG binding affinity can lead to altered total plasma concentrations, with free drug concentrations remaining largely unchanged. However, an increased free fraction can modify the apparent total concentration-effect relationship, confounding the interpretation of therapeutic drug monitoring data which are based on total plasma concentrations [17]. Thus, for drugs such as imatinib, it is important to be able to predict the effects of certain pathophysiological conditions competing with normal binding, as free drug concentrations may be significantly elevated despite total concentrations remaining within the therapeutic range [17]. Going forward, once the binding affinities of imatinib in the absence and presence of uremic toxins are available, the effects of elevated AAG levels versus changes in the binding affinity can be reassessed.

During drug development, typically, initial clinical studies only include patients with normal or only mildly impaired kidney function, as chemotherapeutic agents used to treat cancer generally have narrow therapeutic indices, along with potentially serious adverse toxicities. When extrapolating to doses for patients with moderate to severe RI, in addition to accounting for physiological changes relating to the cancer itself (captured by the Sim-Cancer population), changes in CYP3A4 and potentially other CYP enzymes may need to be considered. During the development of the Sim-Cancer population, changes in CYP abundance were assessed ([20] and references within). A meta-analysis combining several studies determining the in vitro expression or activity of six hepatic CYP enzymes (CYP1A2, 2C8, 2C9, 2C19, 2E1 and 3A4) led to inconclusive results for the CYP enzyme expression/activity in cancer subjects relative to healthy volunteers ([20]; references within). However, it was reported that inflammatory cytokines which can alter levels of CYP expression (e.g., IL-6 reduction of CYP3A levels), resulted in a 28% decrease in midazolam metabolic intrinsic clearance in cancer patients following an oral dose [24]. Simulations demonstrated that by accounting for age differences alone, there was a 36% decrease in CL in patients relative to healthy subjects. Furthermore, a meta-analysis of oral clearance values in healthy volunteers versus cancer patients showed similar elimination across several CYP3A4 substrates, including imatinib, everolimus, ibrutinib, midazolam, dastanib and nilotinib [25,26]. Performance verification using probe CYP substrates including midazolam, caffeine, rosiglitazone, S-warfarin, tolbutamide, dextromethorphan and digoxin demonstrated the predictability of the population model and supported the decision not to include changes in CYP abundance values in the Sim-Cancer population [20]. Based on the findings of our study, this appears to be appropriate. However, potential changes in CYP expression should be investigated when accounting for moderate RI in cancer patients. Assessment of comorbidities in a disease population is a process that can be accommodated using PBPK models. Despite the uncertainties and knowledge gaps related to key parameters that may influence drugs exposure in various clinical conditions, PBPK models can be a valuable tool for estimating prospective dose recommendations and efficacy/safety assessment in special populations when clinical data are lacking [27,28,29].

A recent landscape analysis of anticancer agents approved from 2015 to 2019 was conducted to evaluate the inclusion of study participants with CKD and GFR assessment methods used during drug development and subsequent translation to kidney-related safety and dosing data in product labelling [30]. Of the 74 pivotal trials (involving 55 drugs), the median lower limit of GFR required for inclusion was 45 mL/min. Pharmacokinetic analyses were performed in CKD stages 4–5 for only 29% of drugs. The exclusion of patients with chronic kidney disease from cancer drug trials remains an unsolved problem, which prevents the provision of optimal clinical care for these patients, and raises questions of inclusion, diversity and equity. The case study described here illustrates the strengths of a PBPK modelling approach where the complex interplay between drug-related and physiological parameters can be used to assess comorbid conditions in disease populations. Distributions of AAG concentrations specific to a cancer population reflect the variability in the unbound fraction of imatinib which then drives the hepatic metabolism. Furthermore, reductions of CYP enzyme levels, a consequence of RI, can be applied to reflect varying degrees of renal function. The FDA highlighted the application of PBPK models in the 2020 FDA draft RI guidance, including the early use of this approach to support an expanded inclusion of patients with RI in clinical studies. Although the precise dosage regimens of the cancer patients could not be applied in simulations (average doses were used), the PBPK model was able to capture the observed data (when accounting for additional changes evoked by RI) and the simulations support the finding of the clinical study where it was clearly demonstrated that both AAG levels and the degree of renal impairment are key components that contribute to the interpatient variability associated with imatinib kinetics.

## 5. Conclusions

In this study, we have extended the application of a PBPK modelling approach to predict drug PK in patients with advanced malignancies and varying degrees of renal dysfunction and to elucidate the impact of comorbidities in a cancer population, using imatinib as an illustrative example.

## Figures and Tables

**Table 1:** Patient characteristics and dose escalation schema for imatinib and estimated average dose for each group.

Patient Characteristics		CrCL ≥ 60 mL/min		CrCL 40 to 59 mL/min		CrCL 20 to 39 mL/min		CrCL < 20 mL/min	
		Normal		Mild		Moderate		Severe	
		Group A		Group B		Group C		Group D	
Male, n (%) Female, n (%)	29 (48%) 31 (52%)	Dose (mg)	No. of subjects	Dose (mg)	No. of subjects	Dose (mg)	No. of subjects	Dose (mg)	No. of subjects
Age, median (range)	63 y (16–84)	400	4	400	4	200	8	100	2
Race/Ethnicity		600	4	600	9	400	4	200	
White	54 (90%)	800	6	800	9	600	10	400	
African American	5 (8%)					800	0	600	
Other	1 (2%)	8800	14	14200	22	9200	22	800	
								200	2
		629		645		418		100	

**Table 2:** Predicted and observed dose normalized total and unbound exposures and CL/F of imatinib at steady state following multiple oral doses of imatinib in cancer patients with varying degrees of renal impairment.

	Normal			Mild			Moderate			Moderate *		
TOTAL	Cmax	AUC	CL/F	Cmax	AUC	CL/F	Cmax	AUC	CL/F	Cmax	AUC	CL/F
	ng/mL/mg	(ng/mL*h)/mg	L/h	ng/mL/mg	(ng/mL*h)/mg	L/h	ng/mL/mg	(ng/mL*h)/mg	L/h	ng/mL/mg	(ng/mL*h)/mg	L/h
Predicted	8.14	126	11.2	11.3	198	7.57	12.1	216	6.39	10.5	180	7.91
Observed	6.52	114	10.3	10.6	173	7.50	14.6	229	5.60	14.6	229	5.60
P/O	1.25	1.11	1.09	1.07	1.14	1.01	0.83	0.94	1.14	0.72	0.79	1.41
**UNBOUND**	**Cmax,u**	**AUC,u**	**CL/F,u**	**Cmax,u**	**AUC,u**	**CL/F,u**	**Cmax,u**	**AUC,u**	**CL/F,u**	**Cmax,u**	**AUC,u**	**CL/F,u**
	**ng/mL/mg**	(ng/mL*h)/mg	**L/h**	**ng/mL/mg**	(ng/mL*h)/mg	**L/h**	**ng/mL/mg**	(ng/mL*h)/mg	**L/h**	**ng/mL/mg**	(ng/mL*h)/mg	**L/h**
Predicted	0.48	7.18	181	0.41	6.95	181	0.47	8.16	150	0.41	6.78	186
Observed	0.40	7.07	166	0.65	10.55	123	0.69	10.76	119	0.69	10.76	119
P/O	1.19	1.02	1.09	0.63	0.66	1.47	0.68	0.76	1.26	0.60	0.63	1.56

* No reductions of CYP3A4 and CYP2C8 were considered in simulations.

**Table 3:** Predicted and observed relative changes in total and unbound exposures and CL/F of imatinib at steady state following multiple oral doses of imatinib in cancer patients with varying degrees of renal impairment.

	Mild/Normal			Moderate/Normal			Moderate */Normal		
TOTAL	Cmax	AUC	CL/F	Cmax	AUC	CL/F	Cmax	AUC	CL/F
Predicted	1.39	1.57	0.68	1.49	1.71	0.57	1.29	1.43	0.71
Observed	1.63	1.52	0.73	2.24	2.01	0.54	2.24	2.01	0.54
P/O	0.85	1.04	0.93	0.66	0.85	1.05	0.58	0.71	1.30
**UNBOUND**	**Cmax,u**	**AUC,u**	**CL/F,u**	**Cmax,u**	**AUC,u**	**CL/F,u**	**Cmax,u**	**AUC,u**	**CL/F,u**
Predicted	0.85	0.97	1.00	0.98	1.14	0.83	0.85	0.94	1.03
Observed	1.60	1.49	0.74	1.70	1.52	0.72	1.70	1.52	0.72
P/O	0.53	0.65	1.35	0.58	0.75	1.16	0.50	0.62	1.44

* No reductions of CYP3A4 and CYP2C8 were considered in simulations.

## Data Availability

Not applicable.
